# Complete genome sequence of *Roseburia faecis* M72/1^T^

**DOI:** 10.1128/mra.01253-24

**Published:** 2025-01-08

**Authors:** Kazutoshi Murotomi, Dieter M. Tourlousse, Mayu Hamajima, Yuji Sekiguchi

**Affiliations:** 1 Biomedical Research Institute National Institute of Advanced Industrial Science and Technology (AIST), Tsukuba, Japan; Wellesley College Department of Biological Sciences, Wellesley, Massachusetts, USA

**Keywords:** *Roseburia faecis*, complete genome, Oxford Nanopore Technologies

## Abstract

We describe a complete genome sequence of *Roseburia faecis* strain M72/1^T^ (= JCM 17581^T^ = DSM 16840^T^ = NCIMB 14031^T^). The genome assembly consists of a single circular chromosome with a length of 3,385,415 base pairs and was predicted to contain 3,338 genes and encode for 3,180 proteins.

## ANNOUNCEMENT

Members of the genus *Roseburia* are anaerobic Gram-positive bacteria prevalent in the human gastrointestinal tract (1). The genus currently comprises nine species with validly published names (2), including *Roseburia faecis* (3), also known as “*Agathobacter faecis*.” Like other *Roseburia* species, *R. faecis* is capable of producing butyrate through the fermentation of complex polysaccharides (1, 4). Although less well studied than other prominent members of the *Roseburia* genus, particularly *Roseburia intestinalis*, *R. faecis* is generally thought to be a beneficial microorganism, and its depletion in association with disease has been reported (5). *R. faecis* was also demonstrated to produce a bacteriocin-like substance *in vitro* (6) and protect against stress-induced irritable bowel syndrome in rats (7), underscoring its potential as a probiotic. Here, we employed long-read DNA sequencing, using Oxford Nanopore Technologies (ONT), to generate a complete genome sequence of *Roseburia faecis* M72/1^T^ (= JCM 17,581^T^ = DSM 16840^T^ = NCIMB 14031^T^), the authentic type strain of the species (3).

Cells of strain M72/1^T^ were obtained as liquid-dried (L-dried) cultures from the Japan Collection of Microorganisms (JCM) and grown overnight (~20 h) in AccuDia Modified GAM broth (Shimadzu) under an atmosphere of N_2_/CO_2_ (80/20 [vol/vol]) at 37°C. DNA extraction was performed from the overnight-grown culture using the Nanobind CBB kit RT (PacBio) and prepared for sequencing using the ONT’s Native Barcoding Kit (SQK-NBD114.24; ONT, barcode NB01). Sequencing was performed on an R10.4.1 flow cell using a GridION instrument (MinKNOW v24.02.16), at a speed of 400 bases per second and a sampling frequency of 5 kHz. Bioinformatics procedures below used default parameters unless specified. Bases were called using Dorado v0.8.2 (https://github.com/nanoporetech/dorado) with model dna_r10.4.1_e8.2_400bps_sup@v5.0.0. Low-quality (mean quality of <16) and short reads (<1,000 or <5,000 bases) were filtered using fastq-filter v0.3.0 (https://github.com/LUMC/fastq-filter). The reads with a length of >5,000 bases (reads: 291,384; total bases: 2,601,174,907; N50: 9,059; ~768 × coverage) were split into minimally overlapping subsets using Trycycler v0.5.5’s (8) subsample command. The subsetted reads (~33,000 reads and ~295,000,000 total bases per subset) were assembled using Flye v2.9.5 (9), Raven v1.8.3 (10), and miniasm v0.3 + minipolish v0.1.2 (11). A consensus assembly, involving up to three assemblies per assembler, was generated using Trycycler, including trimming and rotating of circular contigs, and polished using Medaka v2.0.1 (https://github.com/nanoporetech/medaka), with flag--bacteria. No plasmids were identified with plassembler v1.6.2 (12), using the reads with a length of >1,000 bases (reads: 1,251,925; total bases: 5,150,615,274; N50: 5,052) and the polished Trycycler assembly as inputs. Annotation of the genome was performed using the NCBI Prokaryotic Genome Annotation Pipeline v6.8 (13).

The genome has a length of 3,385,415 base pairs and a GC content of 42.97% and consists of a single circular chromosome. Completeness and contamination were estimated to be 99.94% and 0.95%, using CheckM2 v1.0.2’s specific neural network model (14). The genome shares > 99.99% average nucleotide identity with a previously reported fragmented draft assembly of strain M72/1^T^ (RefSeq assembly GCF_001406815.1), which is the current species-level representative in the Genome Taxonomy Database (release 220, 15). The genome was predicted to contain 3,338 total genes, encoding for 3,180 proteins, 52 tRNAs, and four complete sets of rRNA genes.

## Data Availability

The genome sequence has been deposited in the GenBank/DDBJ/EMBL database under accession number CP173697; the version described in this paper is the first version. Sequence data can be found at NCBI’s Sequence Read Archive under accession number SRR31300267.
